# Data from proteomic characterization of the role of Snail1 in murine mesenchymal stem cells and 3T3-L1 fibroblasts differentiation

**DOI:** 10.1016/j.dib.2015.07.027

**Published:** 2015-07-31

**Authors:** A. Peláez-García, R. Barderas, M. Mendes, M. Lopez-Lucendo, J.C. Sanchez, A. García de Herreros, J.I. Casal

**Affiliations:** a Department of Cellular and Molecular Medicine, Centro de Investigaciones Biológicas (CIB-CSIC), Madrid, Spain; b Biochemistry and Molecular Biology I Department, Facultad de Ciencias Químicas, Universidad Complutense de Madrid, Madrid, Spain; c Department of Human Protein Sciences, Faculty of Medicine, University of Geneva, Geneva, Switzerland; d IMIM-Hospital del Mar, Barcelona, Spain

**Keywords:** Snail, Mesenchymal cells, Adipogenesis, Obesity, Proteomics, Differentiation, Transcription factors, IL-17

## Abstract

The transcription factor (TF) Snail1 is a major inducer of the epithelial–mesenchymal transition (EMT) during embryonic development and cancer progression. Ectopic expression of Snail in murine mesenchymal stem cells (mMSC) abrogated their differentiation to osteoblasts or adipocytes. We used either stable isotopic metabolic labeling (SILAC) for 3T3-L1 cells or isobaric labeling with tandem mass tags (TMT) for mMSC stably transfected cells with Snail1 or control. We carried out a proteomic analysis on the nuclear fraction since Snail is a nuclear TF that mediates its effects mainly through the regulation of other TFs. Proteomics data have been deposited in ProteomeXchange via the PRIDE partner repository with the dataset identifiers PXD001529 and PXD002157 (Vizcaino et al., 2014) [1]. Data are associated with a research article published in Molecular and Cellular Proteomics (Pelaez-Garcia et al., 2015) [2].

Specifications table

**Table:** 

Subject area	*Biology*
More specific subject area	*Cell differentiation*
Type of data	*Raw and processed mass spectrometry data acquired and Excel table.*
How data was acquired	*Mass spectrometry, data acquired on LTQ-Orbitrap Velos*
Data format	*.raw (Raw files).*
	*.mzxml (Search files from EasyProt Software)*
	*.msf (Search files from Proteome Discoverer Software)*
	*.mgf (peak files)*
Experimental factors	*Nuclear proteins extracts obtained from 3T3-L1 and mMSC cultured cells with the Subcellular Protein Fractionation Kit (Pierce).*
Experimental features	*Mass spectra were acquired on LTQ-Orbitrap Velos mass spectrometers (Thermo-Scientific) in the positive ion mode.*
Data source location	*Madrid, Spain and Geneva, Switzerland.*
Data accessibility	*The quantitative mass spectrometry proteomics data are available with this article and are related to*[2]*and the PRIDE partner repository: PXD001529 and PXD002157.*

Value of the data

• 938 proteins deregulated in 3T3-L1 cells and 319 proteins deregulated by Snail in mMSC were identified and quantified using a false discovery rate (FDR<1%).
• Most of the proteins were down-regulated, 463 and 294 repressed proteins in 3T3-L1 and mMSC cells, respectively.
• The data provide new insights into the ability of Snail1 to repress newly identified transcription factors, cytokines, growth factors and immunomodulators.
• This work provides insight into novel proteins deregulated by Snail that play important roles into the differentiation inhibition of 3T3-L1 and mMSC cells into adipocytes.
• Most of the deregulated novel proteins by Snail were transcription factors (TFs) as Nr2f6, Trip4 or Prrx1.

## 1 Data and experimental design

Fibroblasts are the most abundant cell type in connective tissues, and some subpopulations are able to differentiate to other cell types as adipocytes or osteoblasts. In normal conditions, fibroblasts are in an inactive quiescent state; however fibroblasts activation is induced by several stimuli, including growth factors such as TGF-β in colorectal cancer. Snail1 transcription is up-regulated by this cytokine in fibroblasts. Snail1 levels are modulated by serum and TGFβ. In addition, Snail1 is not only up-regulated by TGF-β, but also induces the transcription of TGFβ, creating a self-activation loop for the production of TGFβ [3]. The transcription factor Snail1 in mesenchymal cells abrogated their differentiation to osteoblasts or adipocytes. In addition, Snail1 knock-down caused a large decrease in the number of bone marrow murine mesenchymal stem cells (MSCs). This depletion is accompanied of an acceleration of their differentiation to osteoblasts or adipocytes. In this way, Snail would exert its effect on maintaining stemness and pluripotency of MSCs. Snail effects on adipogenesis have been proposed to be mediated, among others, by a lack of response to TGFβ [3] or by an apparent inhibition of PPARγ and C/EBPα expression [4]. Similar results were obtained in preventing the differentiation of bone marrow-derived murine mesenchymal stem cells (mMSC) to osteoblasts or adipocytes. Still, the molecular mechanisms underlying the effect of Snail on MSCs differentiation and the blocking of adipogenesis were far from being established. Confluent 3T3-L1 preadipocytes and mMSCs differentiate to adipocytes upon exposure to a cocktail of adipogenic inducers. During adipogenic differentiation Snail expression is almost negligible in 3T3-L1 cells. As a consequence, Snail1 expression plays an inhibitory effect on adipocyte differentiation program, suggesting a functional role for Snail in obesity.

**Fig. 1:**
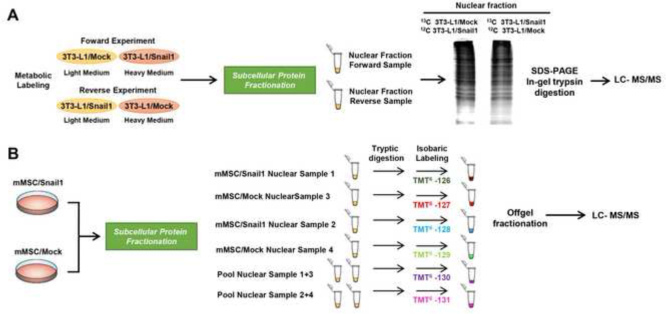
Schematic representation of proteomics experiments. (A) For metabolic labeling with 3T3-L1 cells, 3T3-L1/Snail1 or control cells were grown and separately cultured in light and heavy-labeled DMEM medium supplemented with 10% dialyzed FBS to perform forward and reverse experiments. (B) We performed TMT isobaric labeling for mMSC Snail and control stably transfected cells. mMSC cells were grown in DMEM, detached with PBS containing EDTA 4 mM and subjected to subcellular fractionation. Nuclear protein extracts were labeled to perform two biological and technical replicates in the same experiment.

We carried out an in-depth quantitative proteomic analysis of Snail1-transfected 3T3-L1 and mMSC cells to identify cellular and molecular mechanisms associated to the inhibition of differentiation to adipocytes by Snail. Due to different cell growth requirements, two labeling strategies were followed. Therefore, immortalized 3T3-L1 cells were metabolically labeled in SILAC medium (Fig. 1A). However, since mMSC cells are non-immortal and metabolic labeling requires a high number of duplications for a full isotope incorporation, mMSC cells were subjected to isobaric labeling with tandem mass tags (TMT) (Fig. 1B). We carried out the proteomic analysis on the nuclear fraction since Snail is a nuclear protein that mediates its effects mainly through the regulation of other TFs.

**Fig. 2:**
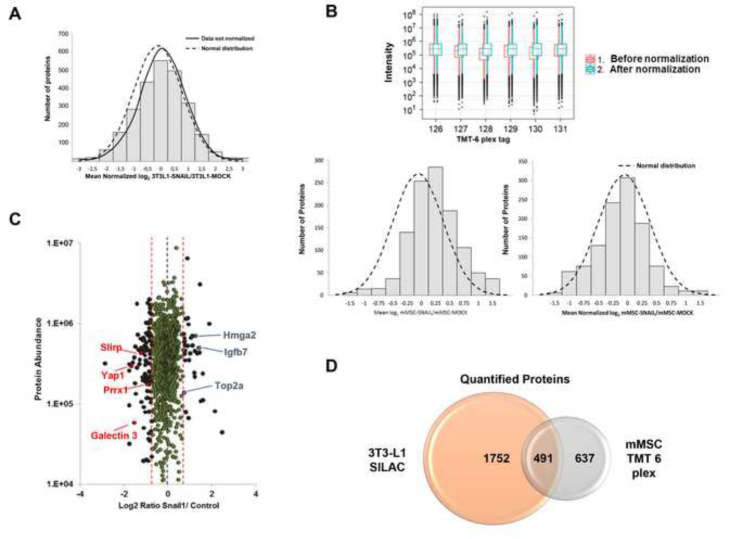
Normalization of proteomics datasets and quantification of proteins in SILAC and TMT proteomic experiments. (A) Histogram plot of the fold-changes for all of the quantified proteins in log_2_-transformed ratios after normalization for both SILAC experiments and the combined data. Data normalization was performed against the 5% trimmed mean to adjust log_2_ protein ratio distribution to zero. Curves representing normal distribution and not normalized data curves are represented. (B) The intensity of the TMT reporters was normalized against the average, which allowed for the optimization of the obtained data from the 6-plex TMT analysis of mMSC cells. Histogram plots of the proteomics data before and after normalization are shown. Curves representing normal distribution are also represented in the figure. (C) Distribution of protein ratios versus protein abundance in Snail1 and control cells by 6-plex TMT analysis. Down-regulated and up-regulated proteins are indicated in black, red, and blue, respectively. Unaltered proteins by Snail are represented in green. (D) Venn diagram showing the coincident quantified proteins in both proteomics SILAC and TMT experiments with 3T3-L1 and mMSC cells.

In Snail-transfected 3T3-L1 cells, we identified 3920 proteins in forward and reverse SILAC experiments, with 2800 overlapping nuclear proteins. In total, 3483 proteins were quantified, with 2251 quantified proteins in common. We normalized the data sets against the 5% trimmed mean to minimize the effect of extreme outliers and to center the protein log_2_ ratio distribution to zero (Fig. 2A). The significance of the fold-change was calculated by a permutation-based statistical test according to p-value [5]. With this fold-change (≥1.5), we found 574 proteins deregulated by Snail1 in the nuclear fraction of 3T3-L1 cells, with 111 and 463 up- and down-regulated proteins, respectively (see Supplementary Tables from [2]).

With TMT, we quantified a total of 1136 proteins in nuclear extracts of mMSCs (Supplementary Table S1 and Supplementary Table S2). TMT6 reporter ion intensities were corrected for isotopic impurities as provided by the manufacturer and normalized imposing equal median intensity (Fig. 2B). We calculated a significant fold-change ≥1.23 by the method of Tan et al. based on random ratios [6]. With this fold-change, we observed 391 deregulated proteins, with 97 and 294 up-regulated and down-regulated proteins, respectively (Fig. 2C). In both proteomic experiment datasets by using DAVID database, we observed a significant enrichment in nuclear proteins with 458 out of 2251 quantified proteins in 3T3-L1 and 531 out of 1136 quantified proteins in mMSC, confirming that the nuclear fraction was correctly obtained. In addition, to confirm the enrichment in nuclear protein, several deregulated proteins were validated in both 3T3-L1 and mMSC cells [2]. In total, 491 proteins were quantified in common in both quantitative SILAC and TMT experiments (Fig. 2D).

This proteomic analysis of Snail effects on adipocyte differentiation in mesenchymal cells identified inhibition of different transcription factors, cytokines or growth factors. Collectively, our results suggest an early effect of Snail on adipogenesis, upstream of C/EBPα and PPARγ proteins, which would be mediated through Nr2f6 and IL-17. We demonstrated the capacity of Snail to induce IL-17 expression in fibroblasts or mesenchymal cells [2].

## 2 Materials and methods

### 2.1 Cell culture and preparation of nuclear cell extracts purification

Preadipocytes 3T3-L1 and murine mesenchymal stem cells (MSCs) stably transfected cells with pcDNA3 Snail1-HA (“snail”) or control pcDNA3 (“mock”) were obtained as indicated [7]. Briefly, stably transfected cells were selected with G418 (geneticin) during 3–4 weeks. Then, stably transfected 3T3-L1 and mMSC cells were grown in DMEM containing 10% FBS, 1 mM l-glutamine, 100 units/ml penicillin–streptomycin, and supplemented with 0.5 µg/ml G418 at 37 °C in 5% CO_2_ [7]. 3T3-L1 and mMSC cells lines were grown in Dulbecco׳s modified Eagle׳s medium (DMEM) (Invitrogen) containing 10% FBS (Biological Industries), 1 mM l-glutamine, and 100 units/ml penicillin–streptomycin and supplemented with 0.5 µg/ml G418 at 37 °C in 5% CO_2_, except for SILAC experiments with 3T3-L1 cells. Cells were resuspended with 4 mM EDTA PBS pH 7.3 and centrifuged, washed with ice-cold PBS prior to subcellular fractionation with the Subcellular Protein Fractionation Kit for Cultured Cells (Pierce) following the manufacturer׳s instructions.

DMEM containing l-lysine and l-arginine or heavy [^13^C_6_]-l-lysine and [^13^C_6_]-l-arginine was purchased from Dundee Cell Products. For metabolic labeling, 3T3-L1 Snail1 or control cells were grown and maintained in DMEM supplemented with 10% dialyzed FBS, 100 units/mL of penicillin/streptomycin at 37 °C in 5% CO_2_, for 8 duplications to achieve >98% incorporation of the heavy amino acids. We carried out forward and reverse experiments to avoid any labeling bias in the study.

### 2.2 SILAC proteomic analysis of 3T3-L1 cells and liquid chromatography tandem mass spectrometry (LC-MS/MS) analysis

Proteins from SILAC labeled nuclear cell extracts of 3T3-L1/Snail and 3T3-L1/Control cells were mixed at a 1:1 ratio and run at 25 mA per gel in 12.5% SDS-PAGE (Fig. 1A). Forward and Reverse experiments as biological and technical replicates were performed to minimize any bias during metabolic labeling. Gels were stained with colloidal Coomassie blue and lanes cut into 18 slices. Excised bands were cut into small pieces and destained with 50 mM ammonium bicarbonate/50% ACN (acetonitrile), dehydrated with ACN and dried. Samples were reduced by adding DTT to a final concentration of 10 mM and alkylated with iodoacetamide to a final concentration of 50 mM. Then, gel pieces were dried, rehydrated with 12.5 ng/µL trypsin in 50 mM ammonium bicarbonate and incubated overnight at 30 °C. Peptides were extracted at 37 °C using ACN and 0.5% TFA, and then dried, cleaned using ZipTip with 0.6 µl C18 resin (Millipore) and reconstituted in 5 µL 0.1% formic acid/2% ACN prior to MS analysis, which was performed as previously described [8].

Peptides were trapped onto a C18-A1 ASY-Column 2 cm precolumn (Thermo-Scientific), and then eluted onto a Biosphere C18 column (C18, inner diameter 75 μm, 10 cm long, 3 μm particle size) (NanoSeparations) and separated using a 170 min gradient from 0% to 35% Buffer B (Buffer A: 0.1% formic acid/2% ACN; Buffer B: 0.1% formic acid in ACN) at a flow-rate of 300 nL/min on a nanoEasy HPLC (Proxeon) coupled to a nanoelectrospay ion source (Proxeon). Mass spectra were acquired on an LTQ-Orbitrap Velos mass spectrometer (Thermo-Scientific) in the positive ion mode. Full-scan MS spectra (*m*/*z* 400–1200) were acquired in the Orbitrap with a target value of 1,000,000 at a resolution of 60,000 at *m*/*z* 400 and the 15 most intense ions were selected for collision induced dissociation (CID) fragmentation in the linear ion trap with a target value of 10,000 and normalized collision energy of 35%. Precursor ion charge state screening and monoisotopic precursor selection were enabled. Singly charged ions and unassigned charge states were rejected. Dynamic exclusion was enabled with a repeat count of 1 and exclusion duration of 30 s. Mass spectra ⁎.raw files were searched against the SwissProt mouse database 57.15 (16,230 sequences) using MASCOT search engine (version 2.3, Matrix Science) through Proteome Discoverer (version 1.4.1.14) (Thermo). Search parameters included a maximum of two missed cleavages allowed, carbamidomethylation of cysteines as a fixed modification and oxidation of methionine, N-terminal acetylation and 13C-Arg, 13C-Lys as variable modifications. Precursor and fragment mass tolerance were set to 10 ppm and 0.8 Da, respectively. Identified peptides were validated using Percolator algorithm with a *q*-value threshold of 0.01 [9]. Relative quantification of identified peptides was performed using Proteome Discoverer with high confidence peptides, Mascot significance threshold of 0.05 and peptide rank=1 filters. For each SILAC pair, Proteome Discoverer determines the area of the extracted ion chromatogram and computes the “heavy/light” ratio. Protein ratios are then calculated as the median of all the unique quantified peptides belonging to a certain protein. The ratios among proteins in their heavy and light versions were used as fold-change. Proteins were quantified with at least one peptide hit in forward and reverse experiments. Deregulated proteins with quantification variability >20% were manually inspected by checking the isotopic envelope of both heavy and light forms and how many peaks of the envelope were used to determine the area of the envelope of all PSMs corresponding to the peptides used to identify the protein. A multipoint normalization strategy was applied to normalize the data sets against the 5% trimmed mean values, which is a robust statistical measure of central tendency that normalize most of the log_2_ protein ratios to 0 [8]. Briefly, the 5% of the most extreme outliers – values – were removed and the mean of the 95% remaining data was determined. This trimmed mean was used to normalize the ratio values, and thus, minimizing the effect of these extreme outliers and centering the log_2_ ratio distribution to zero. Since metabolic conversion arginine/proline can affect quantification accuracy in some cell types, we investigated arginine to proline conversion in 3T3-L1 cells. Using heavy proline as a variable modification, less than 1% of proline-containing peptides were heavy labeled in 3T3-L1 cells.

Proteomics data have been deposited in ProteomeXchange via the PRIDE partner repository with the dataset identifier PXD001529 [1].

### 2.3 Proteomic analysis by isobaric TMT, OFFGEL electrophoresis and liquid chromatography tandem mass spectrometry (LC-MS/MS) analysis

mMSCs were cultured in normal medium, collected and the nuclear fraction obtained. Nuclear protein extracts of mMSC cells were quantified with the Tryptophan method as it was also done for 3T3-L1 cells [10,11]. For nuclear extract of mMSC cells analyzed by TMT, we included in the same proteomic experiment two biological replicates of Snail and control cells and two pools as technical controls (Fig. 1B). In total, 25 μg of proteins from the nuclear extract of each sample were separately dissolved in 33 μl 0.1 M triethylammonium bicarbonate buffer (TEAB) with 6 M urea, reduced with 2 μl 50 mM tris(2-carboxyethyl)phosphine (TCEP) and alkylated with 1 μL of 0.4 M iodoacetamide. In parallel, we performed the same protocol with two other equal tubes containing 25 μg of nuclear protein extracts from each sample (pool sample 1–3 and pool sample 2–4). The samples were then diluted with 64 μl TEAB and digested overnight with 2 μg of porcine trypsin. The next day, all peptides from the four samples and both pools were separately labeled with six different Tandem Mass Tags (Thermo Scientific, San Jose, CA) according to the manufacturer׳s instructions. Finally, the content of the six tubes was pooled, desalted on a Vidac C18 spin column (Harvard apparatus, Holliston, MA) and dried prior to OFFGEL electrophoresis [12].

The TMT experiment was designed to include two biological replicates and two internal controls for normalization. Labeled TMT mMSC samples were separated by OFFGEL according to the manufacturer׳s instructions (Agilent). Briefly, desalted and dehydrated samples were reconstituted in OFFGEL solution. Focusing was performed on an IPG dry strip (13 cm, pH 3–10, linear; GE Healthcare) set up with a 12-well frame at 20 kV h with a maximum of 50 μA and power of 200 mW. The 12 fractions collected were desalted on a Vidac C18 microspin column, dried under speed-vacuum and stored at −20 °C until LC-MS/MS analysis.

ESI LTQ-OT MS was performed on a LTQ Orbitrap velos from Thermo Electron (San Jose, CA, USA) equipped with a NanoAcquity system from Waters as previously described [12]. Peptides were trapped on a home-made 5 µm 200 Å Magic C18 AQ (Michrom) 0.1×20 mm^2^ pre-column and separated on a home-made 5 µm 100 Å Magic C18 AQ (Michrom) 0.75×150 mm^2^ column with a gravity-pulled emitter. The analytical separation was run for 65 min using a gradient of H_2_O/FA 99.9%/0.1% (solvent A) and CH_3_CN/FA 99.9%/0.1% (solvent B). The gradient was run as follows: 0–1 min 95% A and 5% B, then to 65% A and 35% B at 55 min, and 20% A and 80% B at 65 min at a flow rate of 220 nL/min. For MS survey scans, the OT resolution was set to 60,000 and the ion population was set to 5×10E5 with an m/z window from 400 to 2000. A maximum of 3 precursors were selected for both collision-induced dissociation (CID) in the LTQ and high-energy C-trap dissociation (HCD) with analysis in the OT. For MS/MS in the LTQ, the ion population was set to 1×7E3 (isolation width of 2 *m*/*z*) while for MS/MS detection in the OT, it was set to 2×10E5 (isolation width of 2.5 *m*/*z*), with resolution of 7500, first mass at *m*/*z*=100, and maximum injection time of 750 ms. The normalized collision energies were set to 35% for CID and 60% for HCD.

Peak lists were generated from raw data using the embedded software from the instrument vendor (extract_MSN.exe). After peak list generation, the CID and HCD spectra were merged for simultaneous identification and quantification [13]. The monoisotopic masses of the selected precursor ions were corrected using an in-house written Perl script [14]. The corrected mgf files, combined from the 12 analyzed OFFGEL fractions, were searched against the SwissProt mouse database 57.15 (16,230 sequences) using Phenyx (GeneBio, Geneva, Switzerland). The parent ion tolerance was set to 10 ppm and the fragment mass tolerance to 0.8 Da. Variable amino acid modifications were set for oxidized methionine. Trypsin was selected as the enzyme, with two potential missed cleavages, and the normal cleavage mode was used. Only one search round was used with selection of “turbo” scoring. The peptide *p* value was 1×10E−2 for LTQ-Orbitrap data. False-positive ratios were estimated using a reverse decoy database. All datasets where searched once in the forward and once in the reverse database. Separate searches were used to keep the database size constant. Protein and peptide score were then set up to maintain the false positive peptide ratio below 1%. This resulted in a slight overestimation of the false-positive ratio. Only those proteins matching two different peptide sequences were kept for all analyses.

Proteomics data have been deposited in ProteomeXchange via the PRIDE partner repository with the dataset identifier PXD002157 [1].
